# Author Correction: Oscillations of Delta-like1 regulate the balance between differentiation and maintenance of muscle stem cells

**DOI:** 10.1038/s41467-021-22144-w

**Published:** 2021-03-15

**Authors:** Yao Zhang, Ines Lahmann, Katharina Baum, Hiromi Shimojo, Philippos Mourikis, Jana Wolf, Ryoichiro Kageyama, Carmen Birchmeier

**Affiliations:** 1 Developmental Biology/Signal Transduction, Max-Delbrück-Center for Molecular Medicine, Berlin, Germany; 2 Mathematical Modelling of Cellular Processes, Max-Delbrück-Center for Molecular Medicine, Berlin, Germany; 3 Institute for Frontier Life and Medical Sciences, Kyoto University, Kyoto, Japan; 4 Univ Paris Est Creteil, INSERM, IMRB, Creteil, France; 5 Department of Mathematics and Computer Science, Free University Berlin, Berlin, Germany; 6 Neurowissenschaftliches Forschungzentrum, NeuroCure Cluster of Excellence, Charité-Universitätsmedizin Berlin, Berlin, Germany; 7 Present Address: Hasso Plattner Institute, Digital Engineering Faculty, University of Potsdam, Potsdam, Germany; 8 Present Address: Graduate School of Frontier Biosciences, Osaka University, Osaka, Japan

**Keywords:** Cell biology, Developmental biology, Genetics, Stem cells, Systems biology

Correction to: *Nature Communications* 10.1038/s41467-021-21631-4, published online 26 February 2021.

The original version of this Article omitted the following from the Acknowledgements:

This work was supported by a grant by the German Science Foundation, FOR 2841 to C.B.

This has now been corrected in both the PDF and HTML versions of the Article.

