# Supplementary material for: Oscillations of Delta-like1 regulate the balance between differentiation and maintenance of muscle stem cells
Source: Nat Commun. 2021 Feb 26;12:1318. doi: 10.1038/s41467-021-21631-4 (PMC7910593; doi:10.1038/s41467-021-21631-4)
Supplement: Supplementary file 1 — Supplementary Information [file 41467_2021_21631_MOESM1_ESM.pdf]

**Supplementary Information**

# Oscillations of Delta-like1 regulate the balance between differentiation and maintenance of muscle stem cells

Yao Zhang[1#*], Ines Lahmann[1#], Katharina Baum[2,3], Hiromi Shimojo[4,5], Philippos Mourikis[6], Jana Wolf[2,7], Ryoichiro Kageyama[4] and Carmen Birchmeier[1,8]*

**Affiliations:**

[1]Developmental Biology/Signal Transduction, Max-Delbrück-Center for Molecular Medicine, Robert-Rössle-Str. 10, 13125 Berlin, Germany

[2]Mathematical Modelling of Cellular Processes, Max-Delbrück-Center for Molecular Medicine, Robert-Rössle-Str. 10, 13125 Berlin, Germany

[3]New address: Hasso Plattner Institute, Digital Engineering Faculty, University of Potsdam, Prof.-Dr.-Helmert-Str. 2-3, 14482 Potsdam, Germany

[4]Institute for Frontier Life and Medical Sciences, Kyoto University, Kyoto 606-8507, Japan

[5] New address: Graduate School of Frontier Biosciences, Osaka University 1-3 Yamadaoka, Suita, Osaka, 565-0871, Japan

[6]Univ Paris Est Creteil, INSERM, IMRB, F-94010 Creteil, France

[7]Department of Mathematics and Computer Science, Free University Berlin, Arnimallee 14, 14195 Berlin

[8]Neurowissenschaftliches Forschungzentrum, NeuroCure Cluster of Excellence, Charité-Universitätsmedizin Berlin, 10117 Berlin, Germany;

[#]These authors contributed equally; *Corresponding author: cbirch@mdc-berlin.de and Yao.Zhang@mdc-berlin.de

➢ **Supplementary Methods**

**Mathematical modeling of Hes1, MyoD, Dll1 dynamics in single and coupled cells**

➢ **Supplementary Results**

**Supplementary Figure 1. Expression of *Dll1* in muscle regeneration.**

**Supplementary Figure 2. Expression of *Dll1* in developmental limbs and function of Dll1 at early stages of regeneration in the adult muscle.**

**Supplementary Figure 3. Imaging of the oscillatory expression of Dll1 in activated muscle stem cells.**

**Supplementary Figure 4. Defining an enhancer that drives Dll1 expression.**

**Supplementary Figure 5. Oscillatory expression of Dll1 in activated muscle stem cells requires Hes1, whereas MyoD controls robust *Dll1* expression levels.**

**Supplementary Figure 6. Predicted outcome of *Hes1*, *MyoD* and *Dll1*$^{type2}$ mutations on the oscillatory network.**

**Supplementary Figure 7. Effects of the *Dll1*$^{type2}$ mutation on gene expression and oscillatory expression dynamics.**

**Supplementary Figure 8. Effects of the *Dll1*$^{type2}$ mutation on differentiation of cells in cultured spheres and proliferation analysis.**

➢ **Supplementary Table**

➢ **Supplementary References**

## Supplementary Methods: Mathematical modeling of Hes1, MyoD, Dll1 dynamics in single and coupled cells

**Content**

- Modeling the Hes1, MyoD and Dll1 dynamics in single cells using ordinary differential equations (ODEs)
- Modeling Hes1 and Dll1 dynamics with explicit delay in single cells
- Modeling two coupled identical cells with explicit delay
- Modeling two coupled non-identical cells with explicit delay

**Modeling the Hes1, MyoD and Dll1 dynamics in single cells using ordinary differential equations (ODEs)**

We extended a qualitative Hes1-MyoD oscillator model[1,2] to describe the dynamic Hes1, MyoD and Dll1 levels in a single cell. In the original model, *Hes1* mRNA (HR), Hes1 protein (HP), a Hes1 interacting factor (HF), *MyoD* mRNA (MR), MyoD protein (MP) and MyoD interacting factor (MF) are included and it was assumed that Hes1 protein suppresses both *MyoD* and *Hes1* transcription. We extended the model by *Dll1* mRNA (DR) and Dll1 protein (DP) and took into account that *Dll1* transcription is activated by MyoD protein and inhibited by Hes1 protein. The model structure and protein dynamics for the parameters specified below are shown in Fig 5a,b in the main text.

The single-cell ODE model equations are the following:

$$\frac{\mathrm{d\,HP}}{\mathrm{dt}} = k_1 \cdot \mathrm{HR} - k_2 \cdot \mathrm{HP} \cdot \mathrm{HF} - k_3 \cdot \mathrm{HP} \quad (1)$$

$$\frac{\mathrm{d\,HR}}{\mathrm{dt}} = \frac{k_4}{1+\mathrm{HP}^2} - k_5 \cdot \mathrm{HR} \quad (2)$$

$$\frac{\mathrm{d\,HF}}{\mathrm{dt}} = \frac{k_6}{1+\mathrm{HP}^2} - k_2 \cdot \mathrm{HP} \cdot \mathrm{HF} - k_7 \cdot \mathrm{HF} \quad (3)$$

$$\frac{\mathrm{d\,MP}}{\mathrm{dt}} = k_8 \cdot \mathrm{MR} - k_9 \cdot \mathrm{MP} \cdot \mathrm{MF} - k_{10} \cdot \mathrm{MP} \quad (4)$$

$$\frac{\mathrm{d\,MR}}{\mathrm{dt}} = \frac{k_{11}}{1+\mathrm{HP}^2} - k_{12} \cdot \mathrm{MR} \quad (5)$$

$$\frac{\mathrm{d\,MF}}{\mathrm{dt}} = \frac{k_{13}}{1+\mathrm{HP}^2} - k_9 \cdot \mathrm{MP} \cdot \mathrm{MF} - k_{14} \cdot \mathrm{MF} \quad (6)$$

$$\frac{\mathrm{d\,DP}}{\mathrm{dt}} = k_{15} \cdot \mathrm{DR} - k_{16} \cdot \mathrm{DP} \quad (7)$$

$$\frac{\mathrm{d\,DR}}{\mathrm{dt}} = \frac{k_{17}}{1+\mathrm{HP}^2} \cdot \left(k_{19} + \frac{\mathrm{MP}^2}{k_{20}}\right) - k_{18} \cdot \mathrm{DR} \quad (8)$$

For the Hes1- and MyoD-related processes, we used the originally published parameters[2]. $k_1 = 0.3$ and $k_8 = 0.6$ are rate constants of Hes1 and MyoD protein synthesis, respectively. $k_2 = 0.022$ and $k_9 = 0.02$ are rate constants of modulation of Hes1 and MyoD protein degradation by HF and MF, respectively. $k_3 = 0.031$, $k_5 = 0.028$ and $k_7 = 0.3$ are rate constants of degradation of Hes1 protein, *Hes1* transcript and HF (ln2/half-life; half-life = 22.3 min for Hes1 protein; half-life = 24.7 min for *Hes1* mRNA as used in previous models[1,2]). $k_{10} = 0.014$, $k_{12} = 0.0077$,

$k_{14} = 0.03$ are rate constants of degradation of MyoD protein, *MyoD* transcript and MF (ln2/half-life; half-life = 50 min for the MyoD protein[3]; half-life = 90 min for *MyoD* mRNA[4] as used in our previous model[2]). $k_4 = 0.5$ and $k_6 = 20.0$ are rate constants of synthesis of *Hes1* transcripts and HF. $k_{11} = 0.05$ and $k_{13} = 4.0$ are rate constants of synthesis of *MyoD* transcripts and MF. $k_{15} = 0.1$ and $k_{17} = 0.02$ are rate constants of synthesis of Dll1 protein and mRNA, respectively. $k_{16} = 0.017$ and $k_{18} = 0.0231$ are rate constants of degradation of Dll1 protein and *Dll1* mRNA (ln2/half-life; half-life = 40 min for Dll1 protein[5,6]; half-life = 30 min for *Dll1* mRNA[7]). Time was described in minutes, concentrations in arbitrary units, the latter we omitted. All parameters carry the unit 1/min, except for $k_{19} = 10$ and $k_{20} = 5$ that account for constitutive and MyoD-mediated *Dll1* mRNA synthesis, respectively, and have no unit.
*MyoD* knockout was simulated by setting *MyoD* mRNA synthesis to zero, $k_{11} = 0$ (see Supplementary Fig. 6a). *Hes1* knockdown was modeled by increasing the *Hes1* mRNA degradation rate constant to 10-fold to simulate the effect of siRNA, $k_5 = 0.28$. *Hes1* knockout was modeled by setting the *Hes1* mRNA synthesis rate constant to zero, $k_4 = 0$ (see Supplementary Fig. 6b). *Hes1* knockout simulations resulted in similar Dll1 dynamics as observed for *Hes1* knockdown.

**Modeling Hes1 and Dll1 dynamics with explicit delay in single cells**
In order to investigate the influence of transcription delays, we established delay differential equation (DDE) models of the system. We first constructed a single-cell DDE model that represents Hes1 protein and Dll1 protein according to the scheme in Supplementary Fig. 6d. It is based on the framework by Shimojo and colleagues[6], reduced to the single-cell case, and extends it by explicitly modeling Dll1.

In our established single-cell DDE model, Hes1 protein (HP) inhibits synthesis of itself (with delay time $\tau_1$) as well as Dll1 protein (DP) production (with delay time $\tau_{21}$). Both proteins are degraded linearly. The model is governed by the following equation system:

$$\frac{\mathrm{d\,HP}}{\mathrm{dt}}(t) = k_1 \cdot \frac{k_2^2}{k_2^2 + (\mathrm{HP}(t-\tau_1))^2} - k_3 \cdot \mathrm{HP}(t) \quad (9)$$

$$\frac{\mathrm{d\,DP}}{\mathrm{dt}}(t) = k_4 \cdot \frac{k_5^2}{k_5^2 + (\mathrm{HP}(t-\tau_{21}))^2} - k_6 \cdot \mathrm{DP}(t) \quad (10)$$

We successfully mapped the dynamics of the single-cell DDE model to that of our single-cell ODE model (Eq. (1)-(8), Supplementary Fig. 6e). The resulting parameter values that we use unless stated otherwise are the following: Hes1 protein synthesis rate constant $k_1 = 100$, $k_2 = 1^6$, $k_3 = 2.6$ (this corresponds to a half-live of Hes1 protein of 16 min and is similar to the values used in earlier ODE and DDE models[1,2,6,8]), $k_4 = 11.2$, $k_5 = 5.5$, $k_6 = 1.04$ (this corresponds to a half-live of Dll1 protein of 40 min, as in the ODE model above and measured in[5,6]), and delays $\tau_1 = 0.725h$ (similar to the values used in earlier models[6,8]), $\tau_{21} = 0.35h$ (derived from mapping to the ODE model dynamics) for wildtype cells. Time was modeled in hours, concentrations in arbitrary units, the latter we omitted. All parameters are given in the unit 1/h, exceptions are $k_2$ and $k_5$ (that have no unit) and the delays that are given in hours.

The DDE single-cell model (Eq. (9)-(10)) can be used to investigate the impact of alterations of $\tau_{21}$, that is the time Hes1 needs to affect Dll1 protein levels which includes the time needed for *Dll1* transcription. In single cells, we simulated the effect of the *Dll1$^{type2}$* mutation that increases the transcription time of *Dll1* by 0.1h by increasing the delay $\tau_{21}$ in the single-cell DDE model by 0.1h. In the model, this leads to altered phase relationships between Hes1 and Dll1 (see Supplementary Fig. 6f). However, it does neither affect the oscillation amplitudes nor the ability of the cell to oscillate.

**Modeling two coupled identical cells with explicit delay**

Next, we established a DDE model of two identical, coupled cells with explicit delays based on the single-cell DDE model (Eq. (9)-(10)). We coupled the cells by signalling via Dll1 and established a DDE two-cell model representing the dynamics of Hes1 protein, Dll1 protein in cell i ($HP_i$, $DP_i$, i=1,2). In this model, each single cell carries the structure of the single-cell DDE model. In addition, Dll1 protein from cell 1 induces Hes1 synthesis in cell 2 and Dll1 protein from cell 2 induces Hes1 production in cell 1 with a delay $\tau_{22}$ and coupling strength $1/k_7$. We chose a linear activation, which, in combination with the intracellular inhibition of Dll1 protein by Hes1, coincides with the regulations suggested in coupled-cell models of Hes1[6,8]. The delay $\tau_2$ in these models corresponds roughly to a combination of our delays $\tau_{21}$ and $\tau_{22}$. Please refer to Fig. 5c of the main text for a sketch of our model

architecture. The DDE model of two identical coupled cells is given by the following equation system:

$$\frac{\mathrm{d\,HP_1}}{\mathrm{dt}}(t) = k_1 \cdot \frac{k_2^2}{k_2^2+(\mathrm{HP_1}(t-\tau_1))^2} \cdot \frac{\mathrm{DP_2}(t-\tau_{22})}{k_7} - k_3 \cdot \mathrm{HP_1}(t) \quad (11)$$

$$\frac{\mathrm{d\,DP_1}}{\mathrm{dt}}(t) = k_4 \cdot \frac{k_5^2}{k_5^2+(\mathrm{HP_1}(t-\tau_{21}))^2} - k_6 \cdot \mathrm{DP_1(t)} \quad (12)$$

$$\frac{\mathrm{d\,HP_2}}{\mathrm{dt}}(t) = k_1 \cdot \frac{k_2^2}{k_2^2+(\mathrm{HP_2(t}-\tau_1))^2} \cdot \frac{\mathrm{DP_1}(t-\tau_{22})}{k_7} - k_3 \cdot \mathrm{HP_2(t)} \quad (13)$$

$$\frac{\mathrm{d\,DP_2}}{\mathrm{dt}}(t) = k_4 \cdot \frac{k_5^2}{k_5^2+(\mathrm{HP_2(t}-\tau_{21}))^2} - k_6 \cdot \mathrm{DP_2(t)} \quad (14)$$

If not stated otherwise, we used parameter values for wildtype cells as in the single model above: Hes1 protein synthesis rate constant $k_1 = 100$, $k_2 = 1$ (as in[6]), $k_3 = 2.6$ (this corresponds to a half-live of Hes1 protein of 16 min and is similar to the values used in earlier ODE and DDE models[1,2,6,8]), $k_4 = 11.2$, $k_5 = 5.5$, $k_6 = 1.04$ (this corresponds to a half-live of Dll1 protein of 40 min, as in the ODE model above and measured in[5,6]), and delays $\tau_1 = 0.725h$ (similar to the values used in earlier models[6,8]), $\tau_{21} = 0.35h$ (estimated by mapping the single-cell DDE model to the single-cell ODE model dynamics) for wildtype cells. In addition, we used $k_7 = 50$ and $\tau_{22} = 1.3h$ (delay estimated from measurements[9]). As before, we modeled coupling of *Dll1$^{type2}$* mutant cells by increasing $\tau_{21}$ by 0.1h, resulting in $\tau_{21} = 0.45h$ in mutant cells. Also in this model, time is given in hours, concentrations in arbitrary units, the latter we omitted. All parameters have the unit 1/h, except for $k_2$, $k_5$ and $k_7$ (that have no unit) and the delays that are given in h. Fig. 5d in the main text shows the simulations of Dll1 dynamics when coupling two wildtype cells (Fig. 5d left) or two Dll1 type2 mutant cells (Fig. 5d middle).

We systematically investigated the system dynamics of the DDE two-cell model for identical cells (Eq. (11)-(14)) for different values of $\tau_{21}$ in a bifurcation diagram (Supplementary Fig. 6g). Therein, the oscillatory amplitudes of Hes1 and Dll1 of the two coupled cells are given. According to this analysis of the model, we find out-of-phase oscillations for two coupled wildtype cells, quenched oscillations for two coupled *Dll1$^{type2}$* mutant cells (see dashed vertical lines in Supplementary Fig. 6g) and in-phase oscillations in case of larger delays, that is for $\tau_{21} > 0.78h$.

**Modeling two coupled non-identical cells with explicit delay**

In order to model the encounter of two non-identical cells, we adapted the DDE two-cell model for identical cells (Eq. (11)-(14)) to allow different parameters for the two modeled cells. The resulting DDE model represents again the dynamics of Hes1 protein, Dll1 protein in cell i ($HP_i$, $DP_i$, i=1,2) and contains twice the number of kinetic and delay parameters of the DDE model of coupling two identical cells. The model is governed by the following equation system:

$$\frac{\mathrm{d\,HP_1}}{\mathrm{dt}}(t) = k_1 \cdot \frac{k_2^2}{k_2^2+(\mathrm{HP_1}(t-\tau_{1.1}))^2} \cdot \frac{\mathrm{DP_2}(t-\tau_{22.1})}{k_7} - k_3 \cdot \mathrm{HP_1}(t) \quad (15)$$

$$\frac{\mathrm{d\,DP_1}}{\mathrm{dt}}(t) = k_4 \cdot \frac{k_5^2}{k_5^2+(\mathrm{HP_1}(t-\tau_{21.1}))^2} - k_6 \cdot \mathrm{DP_1}(t) \quad (16)$$

$$\frac{\mathrm{d\,HP_2}}{\mathrm{dt}}(t) = k_8 \cdot \frac{k_9^2}{k_9^2+(\mathrm{HP_2}(t-\tau_{1.2}))^2} \cdot \frac{\mathrm{DP_1}(t-\tau_{22.2})}{k_{14}} - k_{10} \cdot \mathrm{HP_2}(t) \quad (17)$$

$$\frac{\mathrm{d\,DP_2}}{\mathrm{dt}}(t) = k_{11} \cdot \frac{k_{12}^2}{k_{12}^2+(\mathrm{HP_2}(t-\tau_{21.2}))^2} - k_{13} \cdot \mathrm{DP_2}(t) \quad (18)$$

Parameters used to model the situation of coupling a wildtype cell (cell 1) with a *Dll1*$^{type2}$ mutant cell (cell 2) (see Fig. 5d right panel, main text) are the same as in the DDE two-cell model when coupling two identical cells except for differences in the intra-cellular delays between Hes1 and Dll1 in cell 1, $\tau_{21.1}$, and cell 2, $\tau_{21.2}$. They take values of $\tau_{21.1} = 0.35h$ and $\tau_{21.1} = 0.45h$, respectively. The other parameter values are $k_1 = k_8 = 100$ , $k_2 = k_9 = 1$ , $k_3 = k_{10} = 2.6$ , $k_4 = k_{11} = 11.2$ , $k_5 = k_{12} = 5.5$ , $k_6 = k_{13} = 1.04$ , $k_7 = k_{14} = 50$, and delays $\tau_{1.1} = \tau_{1.2} = 0.725h, \tau_{22.1} = \tau_{22.2} = 1.3h$ . Time is modeled in hours, concentrations in arbitrary units, the latter are omitted. All parameters are given in the unit 1/h. Exceptions are the parameters $k_2$, $k_5$, $k_7$, $k_9$, $k_{12}$, and $k_{14}$ (that have no unit) and the delays that are given in hours.

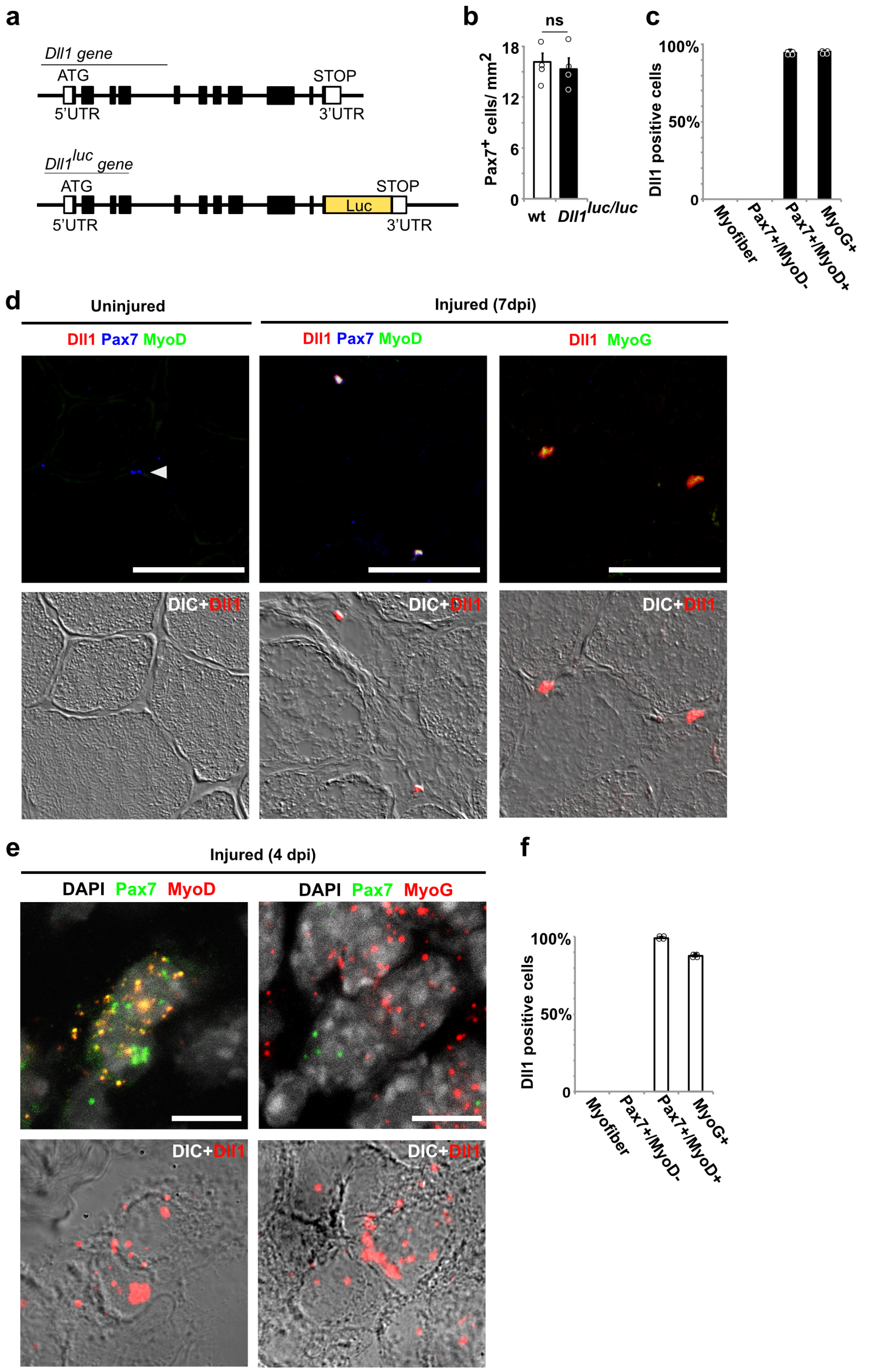


**Supplementary Figure 1. Expression of *Dll1* in muscle regeneration.**

**(a)** Schematic structures of the wildtype *Dll1* and *Dll1*$^{luc}$ alleles; *Dll1*$^{luc}$ encodes a protein in which Dll1 and firefly luciferase are fused. Indicated are the translation initiation (ATG) and stop codons (STOP), 5' and 3' UTRs, exons (black boxes) and the Luc cDNA (yellow box). **(b)** The homozygous *Dll1*$^{luc}$ allele does not interfere with myogenesis, as assessed by a quantification of Pax7+ muscle stem cells in adult

*Dll1*$^{luc/luc}$ animals; n=4 animals. **(c)** Quantification of cells in which Dll1$^{luc}$ protein was detected using anti-luciferase antibodies. Scored were freshly isolated myofibers, the associated quiescent muscle stem cells (Pax7+/MyoD-), and activated (Pax7+/MyoD+) or differentiating (MyoG+) muscle stem cells on myofibers cultured for 72 hrs; n=4 animals. **(d)** smFISH analysis of uninjured and injured muscles (7dpi); the upper panels display signals detected using *Dll1* (red), *Pax7* (blue), *MyoD* or *MyoG* (green) probes as indicated; the lower panels show a differential interference contrast (DIC) image and the *Dll1* signal (red) of the same area; n=3 animals. **(e)** smFISH analysis of the injured muscle (4dpi) showing in the upper panels *Pax7* (green) and *MyoD* or *MyoG* (red) signals; DAPI was used as counterstain. The lower panels show a differential interference contrast (DIC) image and the *Dll1* signal (red) of the same area; n=3 animals **(f)** Quantification of cells displaying *Dll1* smFISH signals. Scored were myofibers and associated quiescent muscle stem cells (Pax7+/MyoD-), as well as activated (Pax7+/MyoD+) and differentiating (MyoG+) muscle stem cells; n=5 animals. Scale bars, 50 μm (d, i) and 10μm (e). Data are presented as mean values +/- SEM; ns indicates P>0.05, unpaired two-sided t-test.

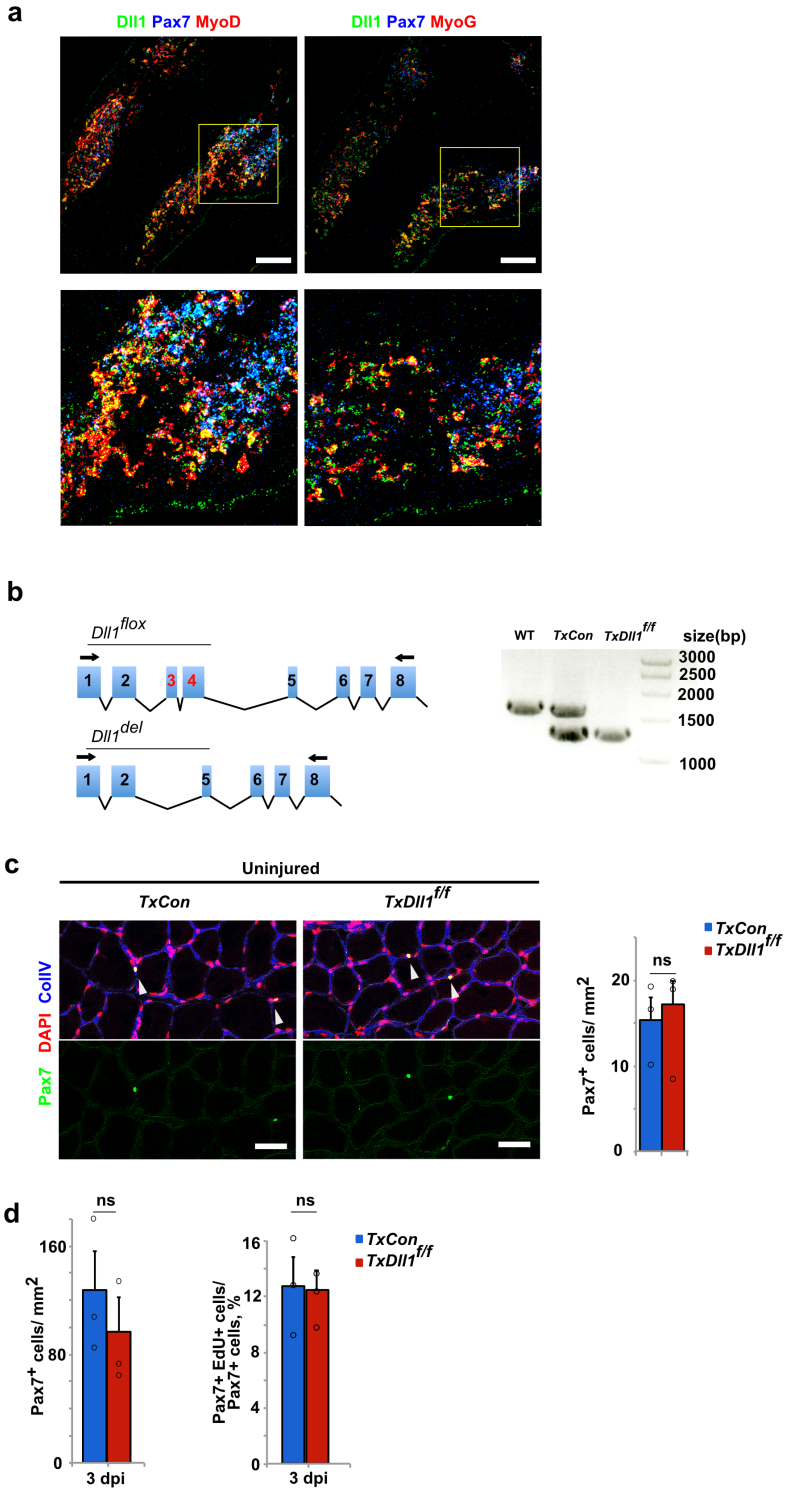


**Supplementary Figure 2. Expression of *Dll1* in developmental limbs and function of Dll1 at early stages of regeneration in the adult muscle.**

**(a)** Expression of *Dll1* in developing limb muscles (E11.5) was assessed by smFISH analysis (E11.5); the upper panels display *Dll1* (green), *Pax7* (blue) and *MyoD* or *MyoG* (red) signals, as indicated. A magnified area is shown below; n=2 animals **(b)**

(left) Schematic structure of the *Dll1*$^{flox}$ allele before and after recombination (upper and lower panels, respectively). Exons are indicated by blue boxes and numbered. Exons 3 and 4 (red numbers) of *Dll1*$^{flox}$ are surrounded by *LoxP* sites. Primers used to detect transcripts are indicated by black arrows. (right) RT-PCR analysis of transcripts isolated from FAC-sorted muscle stem cells; cells were obtained from wildtype, heterozygous (*TxCon*) and homozygous *Dll1*$^{flox}$ (*TxDll1*$^{f/f}$) animals after tamoxifen treatment; n=3 animals. **(c)** The *Dll1* mutation in muscle stem cells does not interfere with the maintenance of the quiescent stem cell pool. (left) Histological analysis of the muscle from control (*TxCon*) and *TxDll1*$^{f/f}$ mutant mice using antibodies against Pax7 (green) and collagen IV (ColIV; blue); DAPI was used as a counterstain (red). (right) Quantification of the number of Pax7+ cells in the uninjured muscle of control (*TxCon*, blue bars) and *TxDll1*$^{f/f}$ (red bars) mice; n=3 animals. **(d)** Quantification of the number of Pax7+ cells and the proliferation of Pax7+ cells (assessed by EdU incorporation) at early stages of muscle regeneration (3dpi) in control (*TxCon*, blue bars) and *TxDll1*$^{f/f}$ (red bars) mutant mice; n=3 animals. Scale bars, 100μm (a) and 50 μm (c). Data are presented as mean values +/- SEM; ns indicates P>0.05, unpaired two-sided t-test.

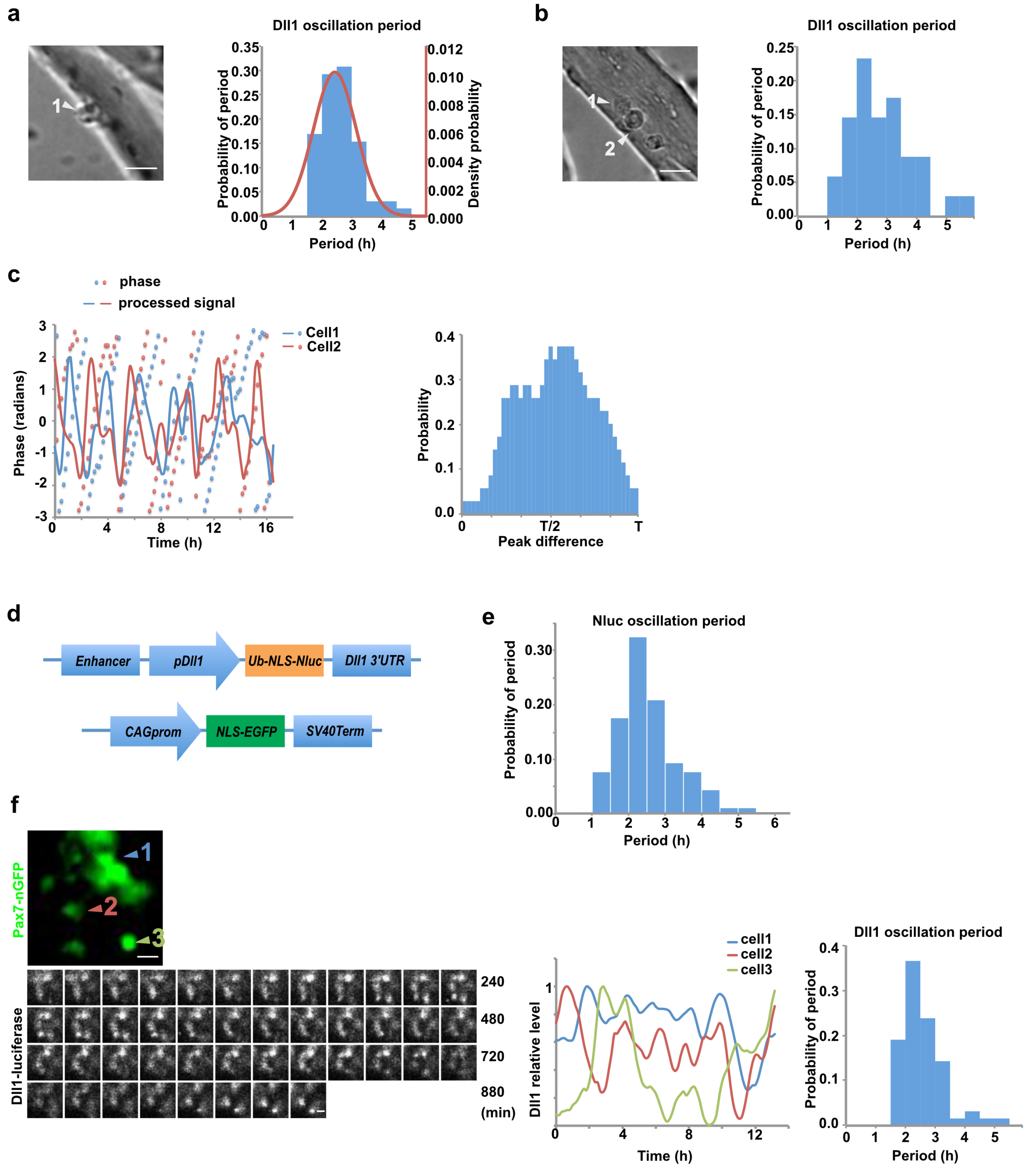


**Supplementary Figure 3. Imaging of the oscillatory expression of Dll1 in activated muscle stem cells.**

**(a)** (left) Brightfield picture of the single activated muscle stem cell associated with a myofiber analyzed in Fig. 2a. (right) Period of Dll1-Luc oscillations observed in single cells associated with myofibers (132 ± 5 mins; n=12 experiments). **(b)** (left) Brightfield picture of the two-cell colony of activated muscle stem cells analyzed in Fig. 2b. (right) Period of Dll1-Luc oscillations in two-cell groups (146 ± 11 mins; n=6

experiments). **(c) (**left) Comparison of Dll1 oscillation phases in two coupled cells on myofibers. (right) Relationship of Dll1 oscillation periods in two coupled cells on myofibers; n=6 experiments. **(d)** Schematic structure of plasmids used to monitor dynamic Dll1 expression in cells cultured in spheres. The *EpDll1-NanoLuc* plasmid (top) encodes a destabilized NanoLuc protein fused to a nuclear localization signal (Ub-NLS-Nluc), whose expression is controlled by the *Dll1* enhancer and promoter. In addition, the 3' UTR of *Dll1* is included. The plasmid encoding nGFP is shown below. **(e)** Period of NanoLuc oscillations in cells grown inside a sphere after transfection of the *EpDll1-NanoLuc* plasmid (127mins ± 4mins; n=13 experiments). **(f)** Analysis of dynamic Dll1 expression in fetal muscle stem cells; the cells were observed in a cultured slice of the limb of an E11.5 $Dll1^{luc}$;$Pax7^{nGFP}$ animal. The GFP signal identified myogenic cells in the slice (upper panel). Below are shown bioluminescence signals observed in these cells (left), the quantification of the bioluminescence signals in three cells indicated by blue, red and green (middle). Quantification of the Dll1 oscillatory period in embryonic cells observed in slices (bottom right); n=3 animals. Scale bars, 15μm.

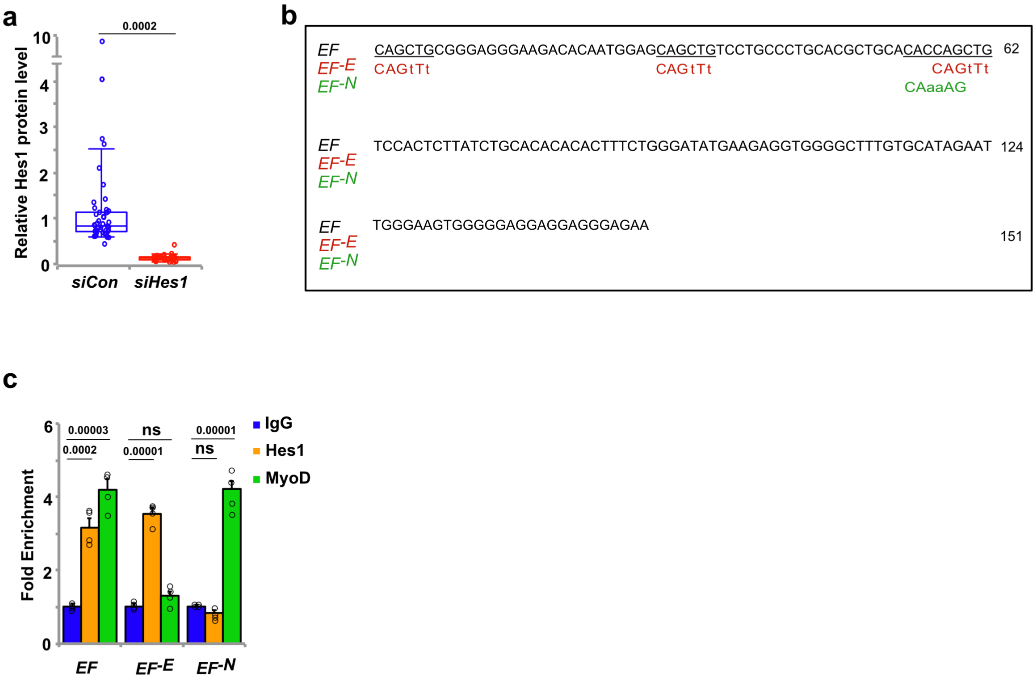


**Supplementary Figure 4. Defining an enhancer that drives Dll1 expression.**

**(a)** Assessment of *Hes1* siRNA efficacy. Relative Hes1 protein levels compared to Pax7 levels in muscle stem cells associated with myofibers treated with control (*siCon*) and *Hes1* (*siHes1*) siRNAs. **(b)** DNA sequence of the *EF* fragment that possesses enhancer activity; the sequences of mutant variants lacking all E-boxes ($EF^{-E}$) or all N-boxes ($EF^{-N}$) are shown below. **(c)** ChIP-PCR analysis of Hes1 and MyoD binding to *EF*, $EF^{-E}$ and $EF^{-N}$ sequences; shown are the fold change of enrichment observed using anti-Hes1 (yellow bars) and anti-MyoD antibodies (green bars) compared to control IgG (blue bars); n=4 experiments. In the box plot, center lines show the medians; box limits indicate the 25th and 75th percentiles; whiskers extend 1.5 times the interquartile range. In the bar plot, data are presented as mean values +/- SEM. Exact p values are indicated, ns indicates P>0.05, unpaired two-sided t-test.

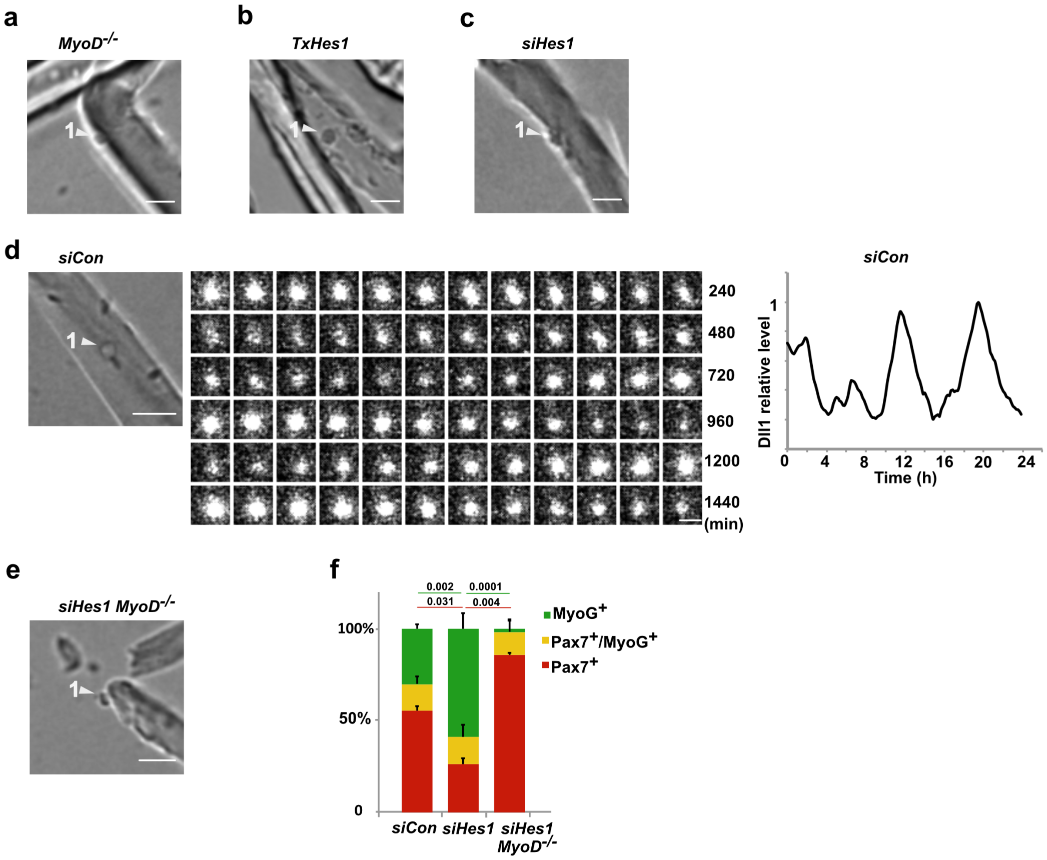


**Supplementary Figure 5. Oscillatory expression of Dll1 in activated muscle stem cells requires Hes1, whereas MyoD controls robust *Dll1* expression levels.**

**(a)** Brightfield picture of the single activated *MyoD*$^{-/-}$ muscle stem cell (arrowhead) associated with a myofiber analyzed in Fig. 4a; the fiber was isolated form a *MyoD*$^{-/-}$; *Dll1*$^{luc}$ animal. **(b)** Brightfield picture of the single activated *Hes1*$^{-/-}$ muscle stem cell (arrowhead) associated with a myofiber analyzed in Fig. 4b; the fiber was isolated form a *TxHes1*;*Dll1*$^{luc}$ animal. **(c)** Brightfield picture of the single activated muscle stem cell (arrowhead) treated with *Hes1* siRNA analyzed in Fig. 4c; the fiber was isolated form a *Dll1*$^{luc}$ animal and imaged after *Hes1* siRNA treatment. **(d)** Brightfield picture (left) of a single activated muscle stem cell (arrowhead) associated with a myofiber and treated with control siRNA; the fiber was isolated form a *Dll1*$^{luc}$ animal and imaged after *siHes1* treatment. Bioluminescence signals observed in this cell (middle) and quantification of the bioluminescence signal (right). **(e)** Brightfield picture of a single activated *MyoD*$^{-/-}$ muscle stem cell treated with *Hes1* siRNA analyzed in Fig. 4d; the fiber was isolated form a *MyoD*$^{-/-}$;*Dll1*$^{luc}$ animal and imaged after *Hes1* siRNA treatment. **(f)** Consequences of *Hes1* siRNA treatment on

differentiation of control and *MyoD*$^{-/-}$ muscle stem cells associated with myofibers; the fibers were analyzed after 72hrs of culture and the relative proportions of MyoG+ (green), Pax7+ (red), Pax7+ and MyoG+ (yellow) cells in colonies on the fibers were determined; n=3 experiments. Scale bars, 15μm. Data are presented as mean values +/- SEM. Exact p values are indicated, unpaired two-sided t-test.

*Supplementary Figure 6*

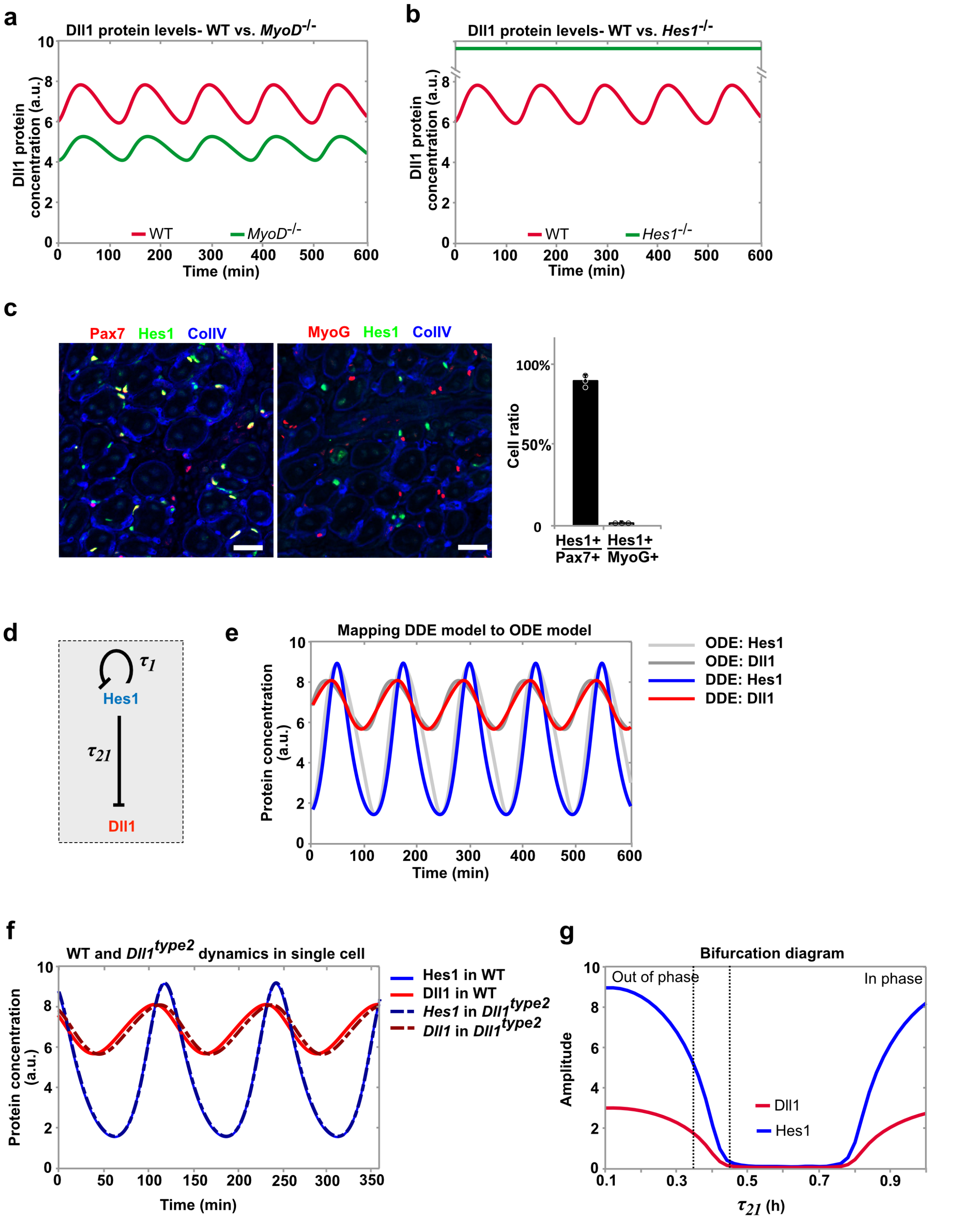


**Supplementary Figure 6: Predicted outcome of *Hes1*, *MyoD* and *Dll1*$^{type2}$ mutations on the oscillatory network and characterization of the delay differential equation model.**

**(a)** Predicted outcome of a *MyoD* mutation on Dll1 expression dynamics; Dll1 protein levels were predicted for wildtype (red) and *MyoD*$^{-/-}$ (green) cells. **(b)** Predicted outcome of a *Hes1* mutation or *Hes1* siRNA treatment on Dll1 expression dynamics;

Dll1 protein levels were predicted for wildtype (red) and *Hes1$^{-/-}$*/*Hes1* siRNA (green) cells. Modeling predictions in (a, b) use the single-cell ordinary differential equation (ODE) model. **(c)** Immunohistological analysis of Hes1 (green), Pax7 or MyoG (red, as indicated) and collagen IV (ColIV, blue) of injured muscle (4 dpi); the quantification (right) demonstrates that MyoG+ cells do not express Hes1; n=3 animals. **(d)** Scheme of regulations and delay times in the single-cell delay differential equation (DDE) model. **(e)** The dynamics in the single-cell DDE model (Hes1: blue, Dll1: red; equations (9)-(10) in Supplementary Methods) can be mapped to the dynamics of the single-cell ODE model (Hes1, light grey and Dll1 dark grey); dynamics in ODE and DDE models are similar for parameter values provided in Supplementary Methods. **(f)** Outcome of a *Dll1$^{type2}$* mutation on expression dynamics in a single cell predicted by the DDE model; dynamics in single wildtype (Hes1, blue solid line; Dll1, red solid line) and *Dll1$^{type2}$* (Hes1, blue stippled line; Dll1, red stippled line) cells are compared. The *Dll1$^{type2}$* mutation was implemented by a 0.1h increase in the delay time $\tau_{21}$. **(g)** Prediction of Hes1 (blue) and Dll1 (red) dynamics in two identical coupled cells using the DDE model (equations (11)-(14)) and varying $\tau_{21}$ values. In this setting, both cells show the same oscillation amplitudes and periods. Zero amplitude denote quenched oscillations. For $\tau_{21} < 0.45$, the two cells oscillate out-of-phase; for values of $\tau_{21}$ between 0.45h and 0.78h, oscillations are severely quenched; for values of $\tau_{21} > 0.78$, cells oscillate in phase. Values that were used for the modeling of coupled wildtype ($\tau_{21} = 0.35$) or *Dll1$^{type2}$* mutant ($\tau_{21} = 0.45$) cells in Fig. 5d are indicated by dotted vertical lines. Data in (c) are presented as mean values +/- SEM. Scale bars, 50 μm.

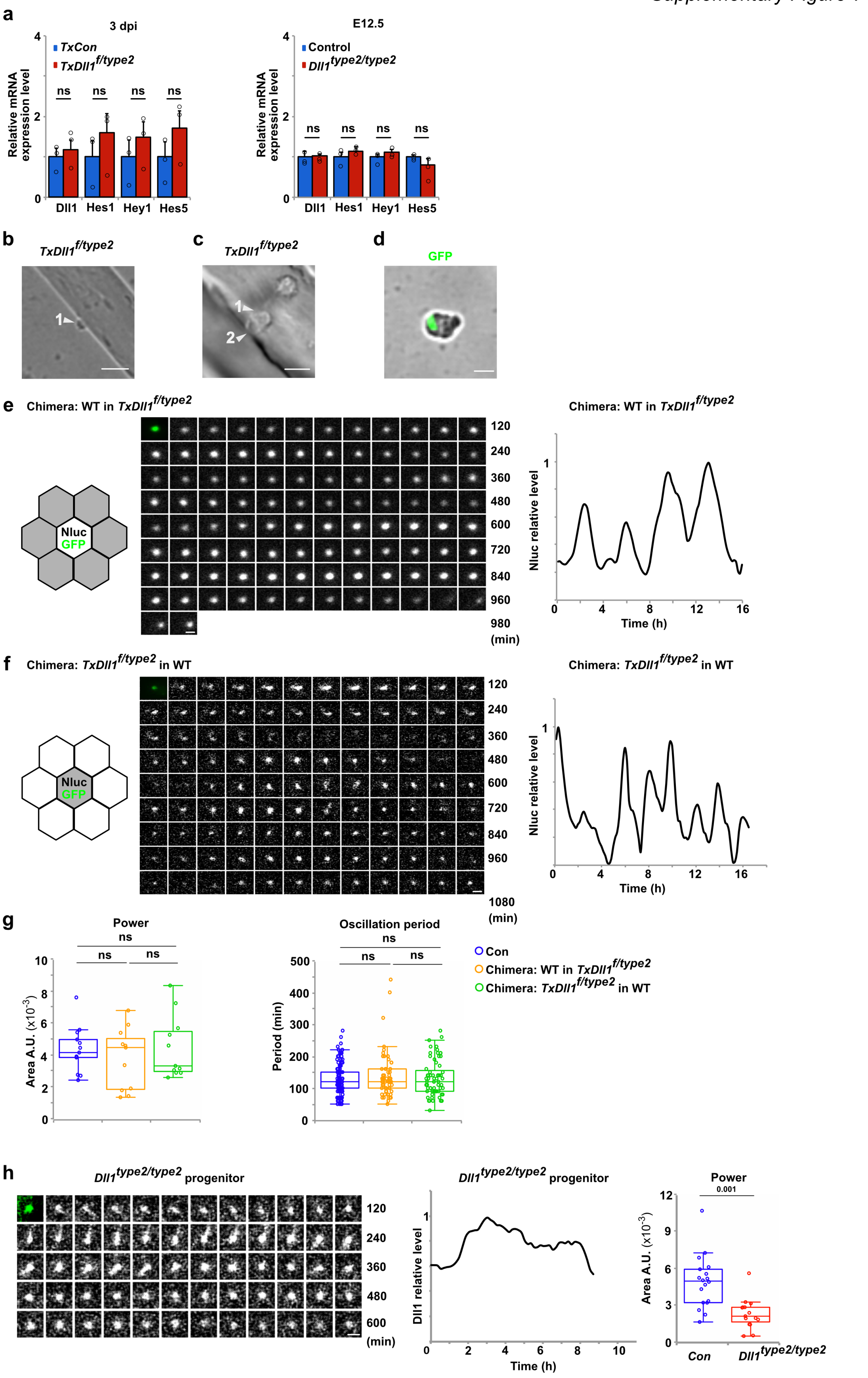
a
3 dpi
TxCon
TxDll1f/type2
Relative mRNA expression level
ns
Dll1
Hes1
Hey1
Hes5
E12.5
Control
Dll1type2/type2
b
TxDll1f/type2
c
TxDll1f/type2
d
GFP
e
Chimera: WT in TxDll1f/type2
Nluc
GFP
(min)
Nluc relative level
Time (h)
f
Chimera: TxDll1f/type2 in WT
g
Power
Area A.U. (x10-3)
Oscillation period
Period (min)
Con
Chimera: WT in TxDll1f/type2
Chimera: TxDll1f/type2 in WT
h
Dll1type2/type2 progenitor
Dll1 relative level
Power
0.001
Con
Dll1type2/type2

**Supplementary Figure 7. Effects of the *Dll1*$^{type2}$ mutation on gene expression and oscillatory expression dynamics.**

**(a)** Expression of *Dll1*, *Hes1*, *Hey* and *Hes5* in muscle stem cells isolated from control (*TxCon*, blue bars) and *Dll1*$^{type2}$ (*TxDll1*$^{f/type2}$, red bars) regenerating (3 dpi) muscle (left), or from control (blue bars) and *Dll1*$^{type2}$ (*Dll1*$^{type2/type2}$, red bars) embryos (E12.5) (right); n=3 animals. **(b)** Brightfield picture of the single fiber-associated *Dll1*$^{type2}$ muscle stem cell analyzed Fig. 6b. **(c)** Brightfield picture of a two-cell colony of *Dll1*$^{type2}$ muscle stem cells on a fiber analyzed in Fig. 6c. **(d)** Brightfield picture of a sphere of *Dll1*$^{type2}$ muscle stem cells analyzed in Fig. 6d. **(e)** Schematic picture of a chimeric sphere showing one wildtype cell (white hexagon) surrounded by *TxDll1*$^{f/type2}$ cells (grey hexagons) (left). Nluc/GFP indicates the wildtype cell transfected with *EpDll1-NanoLuc* and *nGFP* expression plasmids. Dynamic Nanoluc signals in a wildtype cell surrounded by *Dll1*$^{type2}$ cells (middle), and quantification of these signals (right). **(f)** Schematic picture of a chimeric sphere showing one *TxDll1*$^{f/type2}$ cell (grey hexagon) that is transfected with *EpDll1-NanoLuc* and *nGFP* plasmids (Nluc/GFP) and that is surrounded by wildtype cells (white hexagons) (left). Example of dynamic Nanoluc signals in a *TxDll1*$^{f/type2}$ cell surrounded by wildtype cells (middle), and quantification of these signals (right). **(g)** Quantification of the oscillatory stability (left, power of FFT) and oscillatory period in sphere cultures. Compared are spheres containing only wildtype cells (Con: blue) and two chimeric situations, a wildtype cell surrounded by *Dll1*$^{type2}$ cells (wt in *TxDll1*$^{f/type2}$: yellow), and a *Dll1*$^{type2}$ cell surrounded by wildtype cells (*TxDll1*$^{f/type2}$ in wt: green); n=11 experiments. **(h)** Dynamic luciferase signals in muscle stem cells of a cultured slice of a *Dll1*$^{type2/type2}$ embryo (E11.5) (left), and quantification of the signal (middle). Quantification of the oscillatory stability (left, power of FFT) in cells from control (*Con*, blue) and *Dll1*$^{type2/ type2}$ (red) animals; n=3 animals. Scale bars, 15μm. In the box plot, center lines show the medians; box limits indicate 25th and 75th percentiles; whiskers extend 1.5 times the interquartile range. In bar plot, data are presented as mean values +/- SEM. Exact p values are indicated, ns indicates P>0.05, unpaired two-sided t-test.

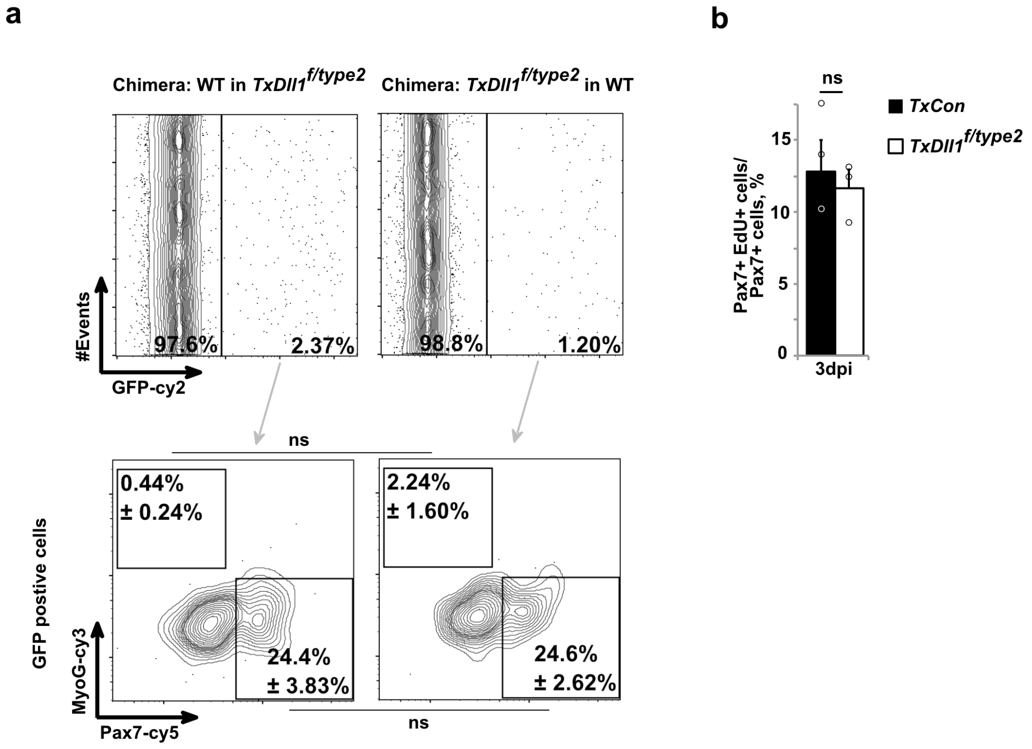


**Supplementary Figure 8. Effects of the *Dll1*$^{type2}$ mutation on differentiation of cells in cultured spheres and proliferation analysis.**

**(a)** FACS gating used for the quantification of MyoG+ and Pax7+ cells in cultured chimeric spheres containing either GFP+ wildtype cells surrounded by GFP-negative *Dll1*$^{type2}$ mutant cells (left) or GFP+ *Dll1*$^{type2}$ mutant cells surrounded by GFP-negative wildtype cells (right). n=3 experiments. **(b)** Quantification of the proliferation of Pax7+ cells (assessed by EdU incorporation) at an early stage of muscle injury (3dpi) in control (*TxCon*, black bar) and *TxDll1*$^{f/type2}$ (white bar) mice; n=3 experiments. Data are presented as mean values +/- SEM. Exact p values are indicated, ns indicates P>0.05, unpaired two-sided t-test.

**Supplementary Table**

| Antibodies | SOURCE | IDENTIFIER |
|---|---|---|
| Rabbit monoclonal Hes1 (1:500) | Cell Signaling Technology | #11988 |
| Guinea pig polyclonal Pax7 (1:100) | Reference [10] | |
| Mouse monoclonal Pax7 (1:1000) | DSHB | AB_528428 |
| Rabbit polyclonal Laminin (1:500) | Abcam | ab14055-50 |
| Goat polyclonal Desmin (1:500) | Santa Cruz | SC-34201 |
| Mouse monoclonal MyoD (5.8A) (1:500) | Santa Cruz | SC-32758 |
| Rabbit polyclonal MyoD (1:500) | Santa Cruz | SC-304 |
| Mouse monoclonal Myogenin (1:500) | ThermoFisher | AB1835 |
| Rabbit polyclonal Myogenin (1:500) | Santa Cruz | SC-576 |
| Goat polyclonal Collagen IV (1:500) | Millipore | AB769 |
| Mouse monoclonal Luciferase (1:500) | DSHB | AB_2722110 |
| Rabbit, goat, mouse, chicken, guinea pig Cy3, Cy2, Cy5 (1:500) | Dianova | |
| DAPI (1:1000) | Sigma-Aldrich | D9542 |
| | | |
| Probes | SOURCE | IDENTIFIER |
| Mm-Dll1-C1 | Bio-Techne | 425071 |
| Mm-Pax7-C2 | Bio-Techne | 314181-C2 |
| Mm-Myod1-C3 | Bio-Techne | 316081-C3 |
| Mm-Myog-C3 | Bio-Techne | 492921-C3 |
| | | |
| Oligonucleotides | SOURCE | IDENTIFIER |
| siRNA targeting Hes1<br>5’-GCTACCCGTAAAGTCCCTA-3’ | This Paper | N/A |
| ChIP-PCR NC1:<br>Forward:<br>5’-GCGTGGCTGTCATTAAGG-3’<br>Reverse:<br>5’-GGTGCTGTCTGCATTACC-3’ | This Paper | N/A |
| ChIP-PCR NC2:<br>Forward:<br>5’-GCCCCGGATTATCGCCTCAC-3’<br>Reverse:<br>5’-TGTCTCCTGCTTTCTGGTTTTGTCTT-3’ | This Paper | N/A |
| ChIP-PCR Mymk:<br>Forward:<br>5’-GAGGCAAGTGCATACCACATGGTAC-3’<br>Reverse:<br>5’-GGACCAGGAGGAAGGCACTGAC-3’ | This Paper | N/A |
| ChIP-PCR MyoD:<br>Forward:<br>5’-GGGTCTTCTCCGGTTTCTCT-3’<br>Reverse:<br>5’-CAATCTCAAAGCCCTGGAAC-3’ | This Paper | N/A |

| | | |
|---|---|---|
| ChIP-PCR Hes1:<br>Forward:<br>5’-GCAGAGAGCAGGTGCTGTCTGCATTACC-3’<br>Reverse:<br>5’-GGGAGATTTTCAACCAACACCACCTTCAC-3’ | This Paper | N/A |
| RT-PCR Myf5:<br>Forward:<br>5’-TGAGGGAACAGGTGGAGAAC-3’<br>Reverse:<br>5’-TGGAGAGAGGGAAGCTGTGT-3’ | This Paper | N/A |
| RT-PCR MyoD:<br>Forward:<br>5’- GCCGCCGCCTGAGCAAAGTGAATG-3’<br>Reverse:<br>5’- GGGGCGCGGCGTCCTGGTC-3’ | This Paper | N/A |
| RT-PCR Dll1:<br>Forward:<br>5’-GCCCCGGATTATCGCCTCAC-3’<br>Reverse:<br>5’-TGTCTCCTGCTTTCTGGTTTTGTCTT-3’ | This Paper | N/A |
| RT-PCR Hes1:<br>Forward:<br>5’- GTGGGTCCTAACGCAGTGTC-3’<br>Reverse:<br>5’- ACAAAGGCGCAATCCAATATG-3’ | This Paper | N/A |
| RT-PCR Hey1:<br>Forward:<br>5’-GCCGACGAGACCGAATCAATAACA-3’<br>Reverse:<br>5’-TCCCGAAACCCCAAACTCCGATAG-3’ | This Paper | N/A |
| RT-PCR Hes5:<br>Forward:<br>5’-GCTCCGCTCGCTAATCGCCTCCAG-3’<br>Reverse:<br>5’-GTCCCGACGCATCTTCTCCACCAC-3’ | This Paper | N/A |
| RT-PCR ß-Actin<br>Forward:<br>5’-GTCCACACCCGCCACCAGTTC-3’<br>Reverse:<br>5’-GGCCTCGTCACCCACATAG-3’ | This Paper | N/A |
| Genotyping Dll1 flox<br>Forward:<br>5’-GCCCCGGATTATCGCCTCAC-3’<br>Reverse:<br>5’-GCCCAAGGGGCAATGGCAGG-3’ | This Paper | N/A |
| | | |
| Plasmids | SOURCE | IDENTIFIER |
| *pGL4.23* | Promega | E841A |
| *pGL-EF* | This Paper | N/A |
| *pGL-EF*$^{-N}$ | This Paper | N/A |

| | | |
|---|---|---|
| *pGL-EF*$^{-E}$ | This Paper | N/A |
| *pCMV-MyoD* | This Paper | N/A |
| *pCMV-MyoG* | This Paper | N/A |
| *phEF1α-Hes1* | Reference [7] | |
| *pCAG-nGFP* | This Paper | N/A |
| *EpDll1-UbNLSNluc-3UTR* | This Paper | N/A |

## Supplementary References


1 Hirata, H. *et al.* Oscillatory expression of the bHLH factor Hes1 regulated by a negative feedback loop. *Science* **298**, 840-843, doi:DOI 10.1126/science.1074560 (2002).

2 Lahmann, I. *et al.* Oscillations of MyoD and Hes1 proteins regulate the maintenance of activated muscle stem cells. *Gene Dev* **33**, 524-535, doi:10.1101/gad.322818.118 (2019).

3 Lingbeck, J. M., Trausch-Azar, J. S., Ciechanover, A. & Schwartz, A. L. Determinants of nuclear and cytoplasmic ubiquitin-mediated degradation of MyoD. *J Biol Chem* **278**, 1817-1823, doi:10.1074/jbc.M208815200 (2003).

4 Figueroa, A. *et al.* Role of HuR in skeletal myogenesis through coordinate regulation of muscle differentiation genes. *Mol Cell Biol* **23**, 4991-5004, doi:10.1128/Mcb.23.14.4991-5004.2003 (2003).

5 Imayoshi, I. *et al.* Oscillatory control of factors determining multipotency and fate in mouse neural progenitors. *Science* **342**, 1203-1208, doi:10.1126/science.1242366 (2013).

6 Shimojo, H. *et al.* Oscillatory control of Delta-like1 in cell interactions regulates dynamic gene expression and tissue morphogenesis. *Gene Dev* **30**, 102-116, doi:10.1101/gad.270785.115 (2016).

7 Shimojo, H., Ohtsuka, T. & Kageyama, R. Oscillations in notch signaling regulate maintenance of neural progenitors. *Neuron* **58**, 52-64, doi:10.1016/j.neuron.2008.02.014 (2008).

8 Yoshioka-Kobayashi, K. *et al.* Coupling delay controls synchronized oscillation in the segmentation clock. *Nature*, doi:10.1038/s41586-019-1882-z (2020).

9 Isomura, A., Ogushi, F., Kori, H. & Kageyama, R. Optogenetic perturbation and bioluminescence imaging to analyze cell-to-cell transfer of oscillatory information. *Gene Dev* **31**, 524-535, doi:10.1101/gad.294546.116 (2017).

10 Brohl, D. *et al.* Colonization of the satellite cell niche by skeletal muscle progenitor cells depends on Notch signals. *Dev Cell* **23**, 469-481, doi:10.1016/j.devcel.2012.07.014 (2012).
