# Supplementary material for: Oscillations of Delta-like1 regulate the balance between differentiation and maintenance of muscle stem cells
Source: Nat Commun. 2021 Feb 26;12:1318. doi: 10.1038/s41467-021-21631-4 (PMC7910593; doi:10.1038/s41467-021-21631-4)
Supplement: Supplementary file 8 — Reporting Summary [file 41467_2021_21631_MOESM8_ESM.pdf]

nature research

Corresponding author(s): Carmen Birchmeier and Yao Zhang

Last updated by author(s): Jan 28, 2021

Reporting Summary

Nature Research wishes to improve the reproducibility of the work that we publish. This form provides structure for consistency and transparency in reporting. For further information on Nature Research policies, see our Editorial Policies and the Editorial Policy Checklist.

Statistics

For all statistical analyses, confirm that the following items are present in the figure legend, table legend, main text, or Methods section.

n/a	Confirmed	
☐	☒	The exact sample size (n) for each experimental group/condition, given as a discrete number and unit of measurement
☐	☒	A statement on whether measurements were taken from distinct samples or whether the same sample was measured repeatedly
☐	☒	The statistical test(s) used AND whether they are one- or two-sided<br>*Only common tests should be described solely by name; describe more complex techniques in the Methods section.*
☒	☐	A description of all covariates tested
☐	☒	A description of any assumptions or corrections, such as tests of normality and adjustment for multiple comparisons
☐	☒	A full description of the statistical parameters including central tendency (e.g. means) or other basic estimates (e.g. regression coefficient) AND variation (e.g. standard deviation) or associated estimates of uncertainty (e.g. confidence intervals)
☐	☒	For null hypothesis testing, the test statistic (e.g. F, t, r) with confidence intervals, effect sizes, degrees of freedom and P value noted<br>*Give P values as exact values whenever suitable.*
☒	☐	For Bayesian analysis, information on the choice of priors and Markov chain Monte Carlo settings
☒	☐	For hierarchical and complex designs, identification of the appropriate level for tests and full reporting of outcomes
☒	☐	Estimates of effect sizes (e.g. Cohen's d, Pearson's r), indicating how they were calculated

Our web collection on statistics for biologists contains articles on many of the points above.

Software and code

Policy information about availability of computer code

Data collection	We used the software ZEN by Zeiss LSM700, cellSens by Olympus IX83 for data collection.
Data analysis	Image processing and analysis were performed using ImageJ(Fiji) and Matlab. The codes will be deposited to Github. The statistics analysis was performed using OriginLab and Excel.

For manuscripts utilizing custom algorithms or software that are central to the research but not yet described in published literature, software must be made available to editors and reviewers. We strongly encourage code deposition in a community repository (e.g. GitHub). See the Nature Research guidelines for submitting code & software for further information.

Data

Policy information about availability of data

All manuscripts must include a data availability statement. This statement should provide the following information, where applicable:

- Accession codes, unique identifiers, or web links for publicly available datasets
- A list of figures that have associated raw data
- A description of any restrictions on data availability

Raw data for the figures xxx are provided in the supplementary dataset.

Field-specific reporting

Life sciences study design

All studies must disclose on these points even when the disclosure is negative.

Sample size	Sample sizes were not pre-determined. We ensured that inclusion of additional data points did not significantly change the variance of the data.
Data exclusions	We did not exclude data.
Replication	To ensure the reproducibility of our findings, we carried out all experiments several independent repeats with similar results. For animal experiments, three or more animals were used. Exact n for each experiment reported in the figure legends.
Randomization	All allocations were random.
Blinding	Investigators were blinded during data analysis.

Reporting for specific materials, systems and methods

We require information from authors about some types of materials, experimental systems and methods used in many studies. Here, indicate whether each material, system or method listed is relevant to your study. If you are not sure if a list item applies to your research, read the appropriate section before selecting a response.

Materials & experimental systems

n/a	Involved in the study	
☐	☒	Antibodies
☐	☒	Eukaryotic cell lines
☒	☐	Palaeontology and archaeology
☐	☒	Animals and other organisms
☒	☐	Human research participants
☒	☐	Clinical data
☒	☐	Dual use research of concern

Methods

n/a	Involved in the study	
☒	☐	ChIP-seq
☐	☒	Flow cytometry
☒	☐	MRI-based neuroimaging

Antibodies

Antibodies used	The details are in the Table S1. Hes1 (Cell signaling 11988), Pax7 (DSHB ab528428 and home made), Laminin (Abcam ab14055-50), Desmin (Santa Cruz sc34201), MyoD (Santa Cruz sc32758 and sc304), Myogenin (Santa Cruz sc576 and ThermoFisher ab1835), CollagenIV (Millipore ab769), Luciferase (DSHB ab2722110)
Validation	All antibodies were validated by the suppliers , with cited references if applicable and accurately represent expected expression patterns. The home made Pax7 antibody was tested on Pax7 mutant tissue.

Eukaryotic cell lines

Policy information about cell lines

Cell line source(s)	C2C12 cells and HEK293 cells were purchased from ATCC (Virginia, US).
Authentication	The differentiation capacity and growth state of C2C12 was checked regularly.
Mycoplasma contamination	All cell lines were negative for mycoplasma contamination.
Commonly misidentified lines (See ICLAC register)	NA

Animals and other organisms

Policy information about studies involving animals; ARRIVE guidelines recommended for reporting animal research

Laboratory animals	All mutant mice in this study had a mixed genetic 129/Sv and C57BL/6 background.
Wild animals	This study did not involve wild animals.
Field-collected samples	This study did not involve field-collected samples.

Ethics oversight	All experiments were conducted according to the policies and regulation established by the Max-Delbrueck-Center for Molecular Medicine (MDC), Germany, and the Mondor Institut of Biomedical Research (IMRB), France.

Note that full information on the approval of the study protocol must also be provided in the manuscript.

Flow Cytometry

Plots

Confirm that:

- [x] The axis labels state the marker and fluorochrome used (e.g. CD4-FITC).
- [x] The axis scales are clearly visible. Include numbers along axes only for bottom left plot of group (a 'group' is an analysis of identical markers).
- [x] All plots are contour plots with outliers or pseudocolor plots.
- [x] A numerical value for number of cells or percentage (with statistics) is provided.

Methodology

Sample preparation	The muscle spheres were dissociated with Trypsin-EDTA as indicated in Methods. The cells were resuspended in 4% PFA for 10min for fixation. Afterwards, they were stained by antibodies as indicated in the Methods.
Instrument	BD FACSAria II with 488, 561, 633 nm lasers
Software	BD FACSDiva Software
Cell population abundance	We used FACSAria II for analysis.
Gating strategy	First we selected viable cells and excluded cell debris by applying FSC-A vs SSC-A gate. We then used FSC-H vs FCS-A to select singlets. Next we used non-primary antibodies stained samples for gate setting.

- [x] Tick this box to confirm that a figure exemplifying the gating strategy is provided in the Supplementary Information.
