# Supplementary material for: Oscillations of Delta-like1 regulate the balance between differentiation and maintenance of muscle stem cells
Source: Nat Commun. 2021 Feb 26;12:1318. doi: 10.1038/s41467-021-21631-4 (PMC7910593; doi:10.1038/s41467-021-21631-4)
Supplement: Supplementary file 9 — Description of Additional Supplementary Files [file 41467_2021_21631_MOESM9_ESM.pdf]

Title: Supplementary Movie 1.
Description: Dll1-luciferase expression in a single muscle stem cell associated with a myofiber, related to Fig. 2a. The fiber was isolated from a mouse carrying the $Dll1^{luc}$ indicator allele. Time-lapse imaging of bioluminescence (left) and bright field (right) signals. Images were taken every 10 minutes; the exposure time for the bioluminescence signal was 6 minutes, and for the bright field image 30 milliseconds.

Title: Supplementary Movie 2.
Description: Dll1-luciferase expression in coupled muscle stem cells associated with a myofiber, related to Fig. 2b. The fiber was isolated from a mouse carrying the $Dll1^{luc}$ indicator allele. Time-lapse imaging of bioluminescence (left) and bright field (right) signals. The arrows (green) show the cells that were tracked. Images were taken every 10 minutes; the exposure time for the bioluminescence signal was 6 minutes, and for the bright field image 30 milliseconds.

Title: Supplementary Movie 3.
Description: Nanoluc expression in a sphere formed by wildtype muscle stem cells, related to Fig. 2c. Time-lapse imaging of bioluminescence (left) and bright field and GFP (right) signals. Images were taken every 10 minutes; the exposure time for the bioluminescence signal was 5 minutes, and for the bright field image 30 milliseconds.

Title: Supplementary Movie 4.
Description: Dll1-luciferase expression in a single muscle stem cell associated with a myofiber, related to Fig. 6b. The fiber was isolated from a $TxDll1^{f/type2}$ mouse. Time-lapse imaging of bioluminescence (left) and bright field (right) signals. Images were taken every 10 minutes; the exposure time for the bioluminescence signal was 9 minutes, and for the bright field image 30 milliseconds.

Title: Supplementary Movie 5.
Description: Dll1-luciferase expression in coupled muscle stem cells associated with a myofiber, related to Fig. 6c. The fiber was isolated from a $TxDll1^{f/type2}$ mouse. Time-lapse imaging of the bioluminescence (left) and bright field (right) signals. The arrows (green) show the cells that were tracked. Images were taken every 10 minutes; the exposure time for the bioluminescence signal was 9 minutes, and for the bright field image 30 milliseconds.

Title: Supplementary Movie 6.
Description: Nanoluc expression in a sphere formed by $TxDll1^{f/type2}$ muscle stem cells, related to Fig. 6d. Time-lapse imaging of a bioluminescence (left) and bright field and GFP (right) signals. Images were taken every 10 minutes; the exposure time for the bioluminescence signal was 5 minutes, and for the bright field image 30 milliseconds.
